# Supplementary material for: The Global Wheat Full Semantic Organ Segmentation (GWFSS) dataset
Source: Plant Phenomics. 2025 Aug 6;7(3):100084. doi: 10.1016/j.plaphe.2025.100084 (PMC12710005; doi:10.1016/j.plaphe.2025.100084)
Supplement: Multimedia component 1 [file mmc1.docx]

# 1 Annex

## 1.1 Data Challenge

By sharing the full GWFSS dataset and tiny set with the annotations, the objective is to enable the computer vision community to design wheat-related segmentation models, including instance segmentation. In particular, the full data set should encourage the development of tools that account for the complex structure of wheat, which can assist in extracting small features in dense vegetation. Recognising the potential of interdisciplinary collaboration in plant phenomics, we propose a data challenge based on the GWFSS dataset. This challenge complements the previous GWHD project, which focused on object detection, by expanding the scope to include segmentation.

This challenge aims to bring together expertise from the phenomics and computer vision communities to explore innovative strategies that mitigate the dependency on large-scale annotations. As pixel-level labelling is both expensive and time-consuming, one of the key objectives of this competition is to explore the potential of leveraging vast unlabelled data to enhance model performance. In this challenge, participants are provided with 65,000 unlabelled images (512×512), cropped from 2,000 full-sized images (Fig. S1). Additionally, a set of 100 labelled images is provided, obtained through stratified sampling from all institutions except Arvalis, which serves as the test set. Mean IOU will be served as the evaluation metric for this competition. By fostering solutions that balance performance and annotation efficiency, the challenge seeks to advance segmentation research while addressing practical limitations. Details of the data challenge can be found via the global wheat website (<https://www.global-wheat.com/gwfss.html>).


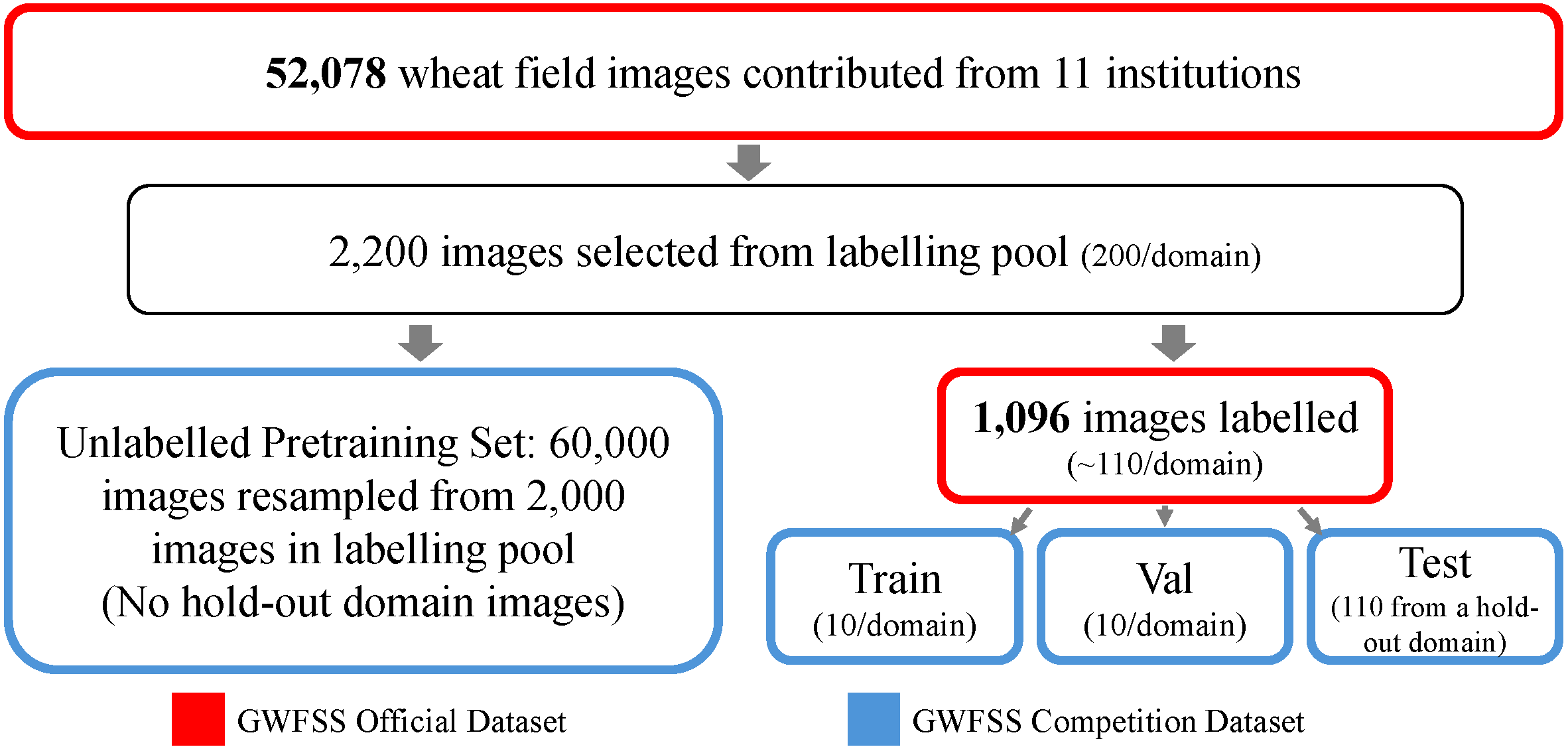


Figure S1: Workflow of dataset selection

Table S1: Summary of all GWFSS variants.


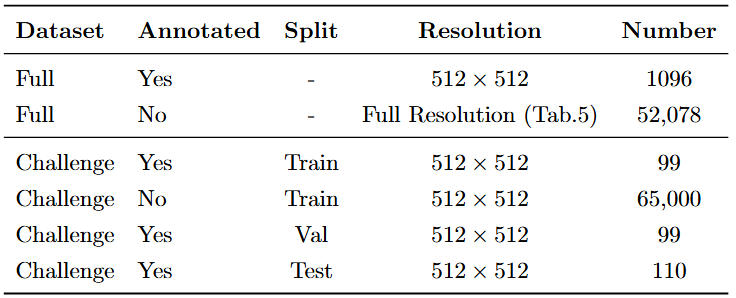


## 1.2 Evaluation of images containing weeds


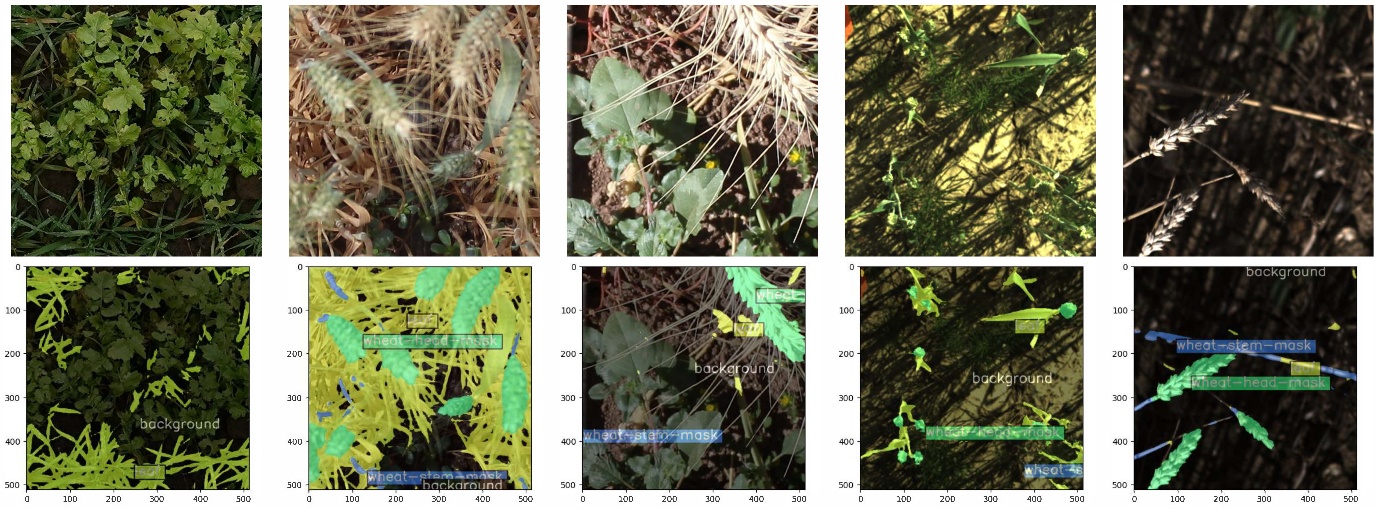


Figure S2: Visualisation of prediction results of all five images that contain weeds in the test set (Random Split, as described in Section 2.5.1)

## 1.3 Evaluation of images during the senescence phase

Table S2: Comparison of Mean Intersection over Union (mIoU) and mean accuracy (mAcc) metrics for Deeplabv3plus (R101) and Segformer (B1) across Random- and Region-based data splits.


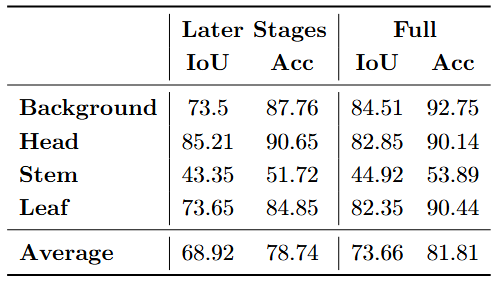


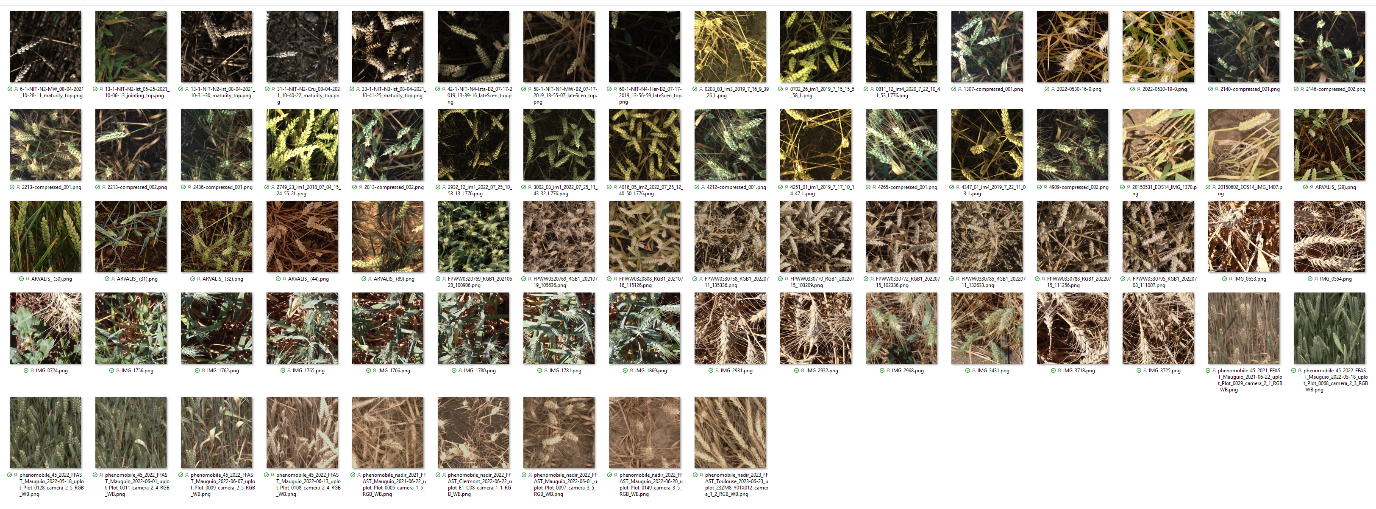


Figure S3: Images of the senescent set selected from the random split (section 2.5.1) to evaluate model performance on canopies with chlorotic and necrotic tissue.
